# Complicações Esofágicas Graves após Ablação de Fibrilação Atrial: Ainda Devemos Nos Preocupar?

**DOI:** 10.36660/abc.20250290

**Published:** 2025-12-18

**Authors:** Anibal Pires Borges, Carlos Kalil, Josep Brugada, José Plutarco Gutiérrez Yanez, Pablo da Costa Soliz, Marco Aurelio Lumertz Saffi, Anderson Donelli da Silveira, Maurício Pimentel

**Affiliations:** 1 Programa de Pós-Graduação em Ciências Cardiovasculares Universidade Federal do Rio Grande do Sul Porto Alegre RS Brasil Programa de Pós-Graduação em Ciências Cardiovasculares da Universidade Federal do Rio Grande do Sul, Porto Alegre, RS – Brasil; 2 Santa Casa de Porto Alegre Porto Alegre RS Brasil Santa Casa de Porto Alegre, Porto Alegre, RS – Brasil; 3 Institut Clínic Cardiovascular Hospital Clínic Universitat de Barcelona Barcelona Espanha Institut Clínic Cardiovascular (ICCV), Hospital Clínic, Universitat de Barcelona, Institut d’Investigacions Biomèdiques August Pi i Sunyer (IDIBAPS), Barcelona – Espanha; 4 Ar-rhythmias Unit Hospital Sant Joan de Déu University of Barcelona Barcelona Espanha Ar-rhythmias Unit, Hospital Sant Joan de Déu, University of Barcelona, Barcelona – Espanha

**Keywords:** Fibrilação Atrial, Fístula Esofágica, Complicações Pós-Operatórias

## Abstract

A ablação da fibrilação atrial (FA) tem sido cada vez mais utilizada como uma estratégia eficaz para o controle do ritmo, resultando na redução da carga arrítmica, melhora da qualidade de vida e, em casos selecionados, diminuição da mortalidade. No entanto, por se tratar de um procedimento invasivo, envolve riscos inerentes. Complicações esofágicas graves, como perfuração esofágica ou fístula átrio-esofágica, embora raras (com incidência variando de 0,025% a 0,113%), estão associadas a significativa morbidade e mortalidade. Diversos fatores de risco para lesão esofágica foram identificados, incluindo características relacionadas tanto ao paciente quanto ao procedimento. Para mitigar esses riscos durante a ablação por radiofrequência (RF), várias medidas preventivas foram adotadas, como o uso de cateteres com controle de força de contato, aplicações de energia de alta potência e curta duração, além de estratégias de proteção como monitoramento da temperatura esofágica, dispositivos de deslocamento e técnicas de resfriamento. Lesões esofágicas profundas detectadas por endoscopia de vigilância são consideradas precursoras de complicações mais graves e, portanto, merecem atenção especial. O monitoramento intensivo de pacientes com essas lesões pode ser crucial para permitir o diagnóstico precoce e a intervenção oportuna, prevenindo a progressão para complicações mais graves. Embora a terapia por ablação com campo pulsado – que apresenta menor risco para o esôfago e outras estruturas adjacentes – ainda não esteja amplamente disponível, a implementação de estratégias eficazes de monitoramento pode melhorar significativamente os desfechos clínicos e aumentar a segurança dos pacientes no contexto da ablação por RF da FA.

## Graphical abstract

**Figura Central:**
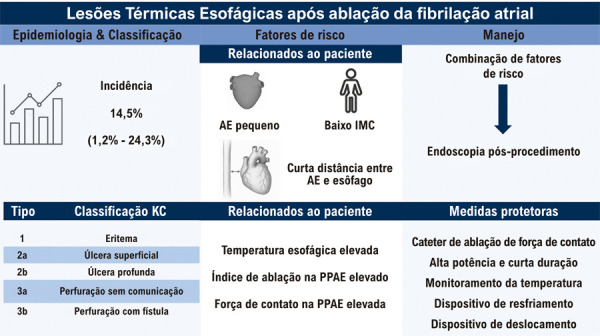
Complicações Esofágicas Graves após Ablação de Fibrilação Atrial: Ainda Devemos Nos Preocupar?

## Introdução

A Fibrilação Atrial (FA) é a arritmia sustentada mais prevalente em adultos, associada à significativa morbidade e mortalidade, além de impor uma carga substancial aos pacientes, profissionais de saúde e sistemas de saúde em todo o mundo.^1^ A ablação por cateter representa uma estratégia terapêutica chave no manejo da FA, com eficácia superior no controle do ritmo em comparação aos medicamentos antiarrítmicos. Ainda, há evidências de melhora dos sintomas, aumento da qualidade de vida^2-4^ e, em pacientes com insuficiência cardíaca e fração de ejeção reduzida, redução da mortalidade.^5,6^

**Figura 1:**
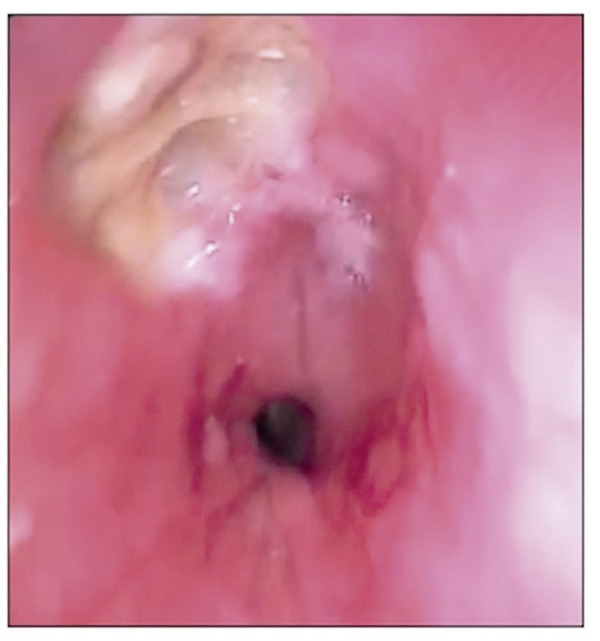
– Perfuração esofágica após ablação da fibrilação atrial em um paciente que desenvolveu uma fístula átrio-esofágica (imagem dos autores).

Apesar de seus benefícios, a ablação da FA envolve riscos relacionados ao acesso vascular, manipulação de cateteres e entrega de energia no Átrio Esquerdo (AE), que está próximo de estruturas sensíveis ao calor. Dados recentes indicam taxas de complicações entre 2,5% e 8%, com mortalidade em 30 dias inferior a 0,1%. A experiência do operador e maiores volumes de procedimentos estão associados a menos complicações.^1,7^ Ao longo dos anos, essas taxas de complicações diminuíram significativamente.^8^ Complicações graves da ablação de FA incluem perfuração esofágica e Fístula Átrio-esofágica (FAE) (Figura 1), eventos tromboembólicos, tamponamento cardíaco, estenose das veias pulmonares, lesão do nervo frênico e complicações vasculares.^1,7^

Embora raras, lesões esofágicas graves – especialmente perfuração e FAE – estão associadas à elevada morbidade e mortalidade.^7^ Compreender os fatores que contribuem para essas complicações é essencial, especialmente considerando o caráter eletivo do procedimento. Essa preocupação nos motivou a realizar uma revisão sobre o tema. A revisão incluiu publicações relevantes indexadas no PubMed entre 2004 e 2025, utilizando as seguintes palavras-chave: ablação da fibrilação atrial, complicações esofágicas, FAE e Lesões Térmicas Esofágicas (LTEs).

### Fístula átrio-esofágica: incidência, fisiopatologia e lesões precursoras

A FAE foi descrita pela primeira vez em 2004, com um caso relatado por Scanavacca et al.^9^ e dois por Pappone et al.^10^ Entre esses casos iniciais, dois pacientes faleceram e um precisou de intervenção cirúrgica de emergência. Um registro brasileiro, abrangendo 8863 procedimentos realizados entre 2003 e 2015, identificou dez casos de FAE, correspondendo a uma incidência de 0,113%. Desses, sete foram fatais e dois sobreviventes apresentaram sequelas neurológicas.^1 1^ No estudo POTTER-AF – o maior registro sobre essa complicação até o momento – foram relatados 138 casos de FAE entre 553 729 ablações, resultando em uma incidência de 0,025%. A taxa de mortalidade foi de 65,8%, com apenas 15,4% dos pacientes se recuperando sem sequelas.^1 2^ Embora a incidência relatada no estudo POTTER-AF seja menor do que a observada no registro brasileiro, ela destaca o impacto clínico devastador dessa complicação, apesar dos avanços tecnológicos nas técnicas de ablação. Além disso, a verdadeira incidência de FAE pode estar subestimada devido às práticas limitadas de rastreamento em muitos centros e à ausência de dados abrangentes da América Latina.^13^

A fisiopatologia precisa da FAE ainda não é completamente compreendida, mas acredita-se que a LTE sustentada durante a ablação do Átrio Esquerdo (AE) inicie a cascata que leva à perfuração esofágica e à formação da fístula. Os mecanismos propostos incluem lesão direta da mucosa causada pela energia térmica, agravada pela doença do refluxo gastroesofágico e pela motilidade esofágica prejudicada, possivelmente devido à lesão do plexo nervoso periesofágico durante o procedimento ou aos efeitos da anestesia geral.^14^

O antro das Veias Pulmonares (VPs), localizado na Parede Posterior do AE (PPAE) e delimitado pelos quatro óstios das VPs, é o principal alvo durante a ablação circunferencial antral ampliada. Essa técnica utiliza energia de radiofrequência para criar bloqueio de condução, induzindo descontinuidade muscular e isolando eletricamente as VPs. No entanto, a extensão das lesões necessária para um isolamento eficaz pode, inadvertidamente, aumentar o risco de dano térmico às estruturas adjacentes, particularmente ao esôfago.^15^

**Figura 2:**
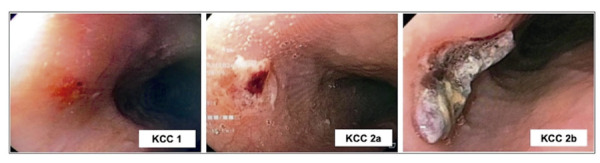
– Exemplos de lesões térmicas esofágicas (imagens dos autores).

A endoscopia digestiva alta pós-procedimento é uma ferramenta valiosa para a identificação precoce do comprometimento esofágico, com incidência relatada de LTEs em séries recentes variando de 1,2% a 24,3%.^16,17^ As LTEs são consideradas lesões precursoras de complicações mais graves. O sistema de classificação mais amplamente adotado para LTEs foi proposto pelo grupo de Kansas City, com base em 30 estudos envolvendo 4473 pacientes, dos quais 3921 foram submetidos à endoscopia ou cápsula endoscópica dentro de sete dias após a ablação da FA. Nesta revisão sistemática, a incidência geral de LTEs foi de 14,5% (570 casos), categorizados da seguinte forma:^18^ tipo 1 – eritema; tipo 2a – úlcera superficial; tipo 2b – úlcera profunda; tipo 3a – perfuração esofágica sem FAE; e tipo 3b – perfuração esofágica com FAE (Tabela 1, Figura 2).

**Tabela 1:** – Classificação Kansas City das lesões esofágicas após a ablação da fibrilação atrial (adaptada de Yarlagadda et al., 2019)

Tipo	Característica
**1**	Eritema
**2a**	Úlcera superficial
**2b**	Úlcera profunda
**3a**	Perfuração sem comunicação com o átrio
**3b**	Perfuração com fístula átrio-esofágica

Entre 428 pacientes com lesões tipo 1 ou 2a, nenhum evoluiu para perfuração ou FAE. Em contraste, entre 142 pacientes com lesões tipo 2b (úlceras profundas), seis desenvolveram perfurações: cinco casos não estavam associados à FAE (um dos quais foi fatal), e um evoluiu para FAE (também fatal). Um registro separado com 1802 pacientes submetidos à ablação da FA e avaliação endoscópica dentro de sete dias após o procedimento relatou uma incidência de 18% de LTEs (150 pacientes). Dentre esses, 98 apresentaram eritema ou erosões, enquanto 52 apresentaram úlceras (lesões profundas). A progressão para perfuração esofágica ou FAE ocorreu em cinco pacientes – todos com úlceras profundas previamente identificadas como lesões precursoras.^19^Esses achados ressaltam a importância da identificação precoce e da vigilância rigorosa de pacientes com lesões esofágicas profundas, que podem se beneficiar de intervenções direcionadas para mitigar o risco de complicações graves.

### Fatores de risco para lesão térmica esofágica

Diversos fatores de risco para o desenvolvimento de lesão esofágica após a ablação da FA foram relatados, embora os achados nem sempre tenham sido consistentes entre os estudos. Alguns desses fatores estão relacionados ao paciente e são pré-existentes, enquanto outros são relacionados ao procedimento e só identificados durante a ablação.

### Fatores de risco relacionados ao paciente

Vários fatores de risco para LTE têm sido associados a características anatômicas do paciente. Em um estudo envolvendo 238 pacientes, uma menor distância entre o AE e o esôfago foi identificada como um preditor significativo de lesão (3,0 mm vs. 4,4 mm; p = 0,012). Além disso, a área do AE, avaliada por Ressonância Magnética (RM) foi menor em pacientes que desenvolveram lesão esofágica (24,92 ± 6,49 cm^2^ vs. 29,53 ± 7,47 cm^2^; p = 0,032), sugerindo que dimensões reduzidas do AE podem estar correlacionadas com espaços anatômicos periesofágicos mais estreitos.^20^

Um estudo baseado em registro realizado por Ferraz et al.,^17^ envolvendo 677 pacientes, demonstrou que um maior diâmetro do AE estava associado a um menor risco de LTE. Um aumento de 1 mm no diâmetro do AE correspondia a uma redução de 7% na incidência de lesão (razão de chances [OR], 0,93; intervalo de confiança de 95% [IC], 0,88–0,99; p = 0,028).^17^ Ainda, em uma coorte de 267 pacientes submetidos à ablação de FA sem monitoramento da temperatura esofágica e com limite de potência de 25 W aplicado à PPAE, uma menor distância entre o AE e o esôfago — avaliada por tomografia computadorizada — também foi associada a um risco aumentado de LTE. A distância média entre o AE e o esôfago nos pacientes que desenvolveram lesão foi de 2,0 ± 0,3 mm, em comparação com 2,5 ± 0,7 mm naqueles sem lesão. A análise multivariada confirmou essa distância como o único preditor significativo de LTE (p = 0,0176).^21^

A associação entre Índice de Massa Corporal (IMC) e LTE também foi investigada. Em um estudo com 104 pacientes submetidos à ablação de FA com potência máxima de 25 W e aplicações de 30 segundos na PPAE, a incidência de lesão esofágica ou de nervo periesofágico assintomática foi de 9,6%. Observou-se uma associação significativa entre IMC baixo e maior risco de lesão, com cada redução de 1 kg/m^2^ no IMC correspondendo a um aumento de 24% no risco (razão de chances [OR], 0,76; intervalo de confiança [IC] de 95%, 0,59–0,97; P < 0,05). Todos os pacientes afetados tinham IMC inferior a 24,9 kg/m^2^.^22^

Em contraste, outro estudo não encontrou associação entre baixo IMC e lesão esofágica em uma coorte de 20 pacientes com IMC < 24,9 Kg/m^2^ submetidos à ablação utilizando energia limitada (máximo de 20 W na parede posterior do átrio esquerdo), curta duração (máximo de 20 segundos) e temperatura esofágica controlada (≤39°C).^23^ Mais recentemente, um estudo randomizado comparando dois tipos de sondas multissensoriais esofágicas de temperatura relatou uma incidência de 6,6% de LTE, com IMC significativamente menor nos pacientes que sofreram lesão em comparação com aqueles que não sofreram (21,4 ± 2,5 Kg/m^2^ vs. 24,3 ± 3,4 Kg/m^2^; p = 0,022).^24^

Esses achados sugerem que certas características dos pacientes, identificáveis antes do procedimento, podem predispor a um risco aumentado de lesão esofágica. Pacientes com IMC mais baixo e aqueles com AE de tamanho menor ou anatomicamente normal podem apresentar maior risco de LTE durante a ablação da FA.

### Fatores de risco relacionados ao procedimento

Além dos fatores relacionados ao paciente, diversas variáveis relacionadas ao procedimento têm sido associadas a um risco aumentado de LTE durante a ablação da FA.

A elevação da temperatura esofágica foi identificada como um fator de risco significativo em pacientes submetidos à ablação por Radiofrequência (RF) com monitoramento da temperatura esofágica. Em um estudo realizado por Halbfass et al.,^19^ o monitoramento da temperatura esofágica foi utilizado em 35% dos 832 procedimentos de ablação de FA. Uma temperatura esofágica máxima superior a 40,5 °C foi um preditor independente de lesão esofágica [razão de chances (OR) 4,044; intervalo de confiança (IC) de 95%, 2,049–7,984; p < 0,001]. Além disso, quando esse limite de temperatura foi atingido, o risco relativo (RR) para o desenvolvimento de úlceras profundas aumentou para 2,76.^19^

Em uma coorte de 88 pacientes submetidos à ablação de FA) com limitação de potência guiada por temperatura durante as lesões na PPAE, temperaturas ≥ 42 °C foram associadas a uma maior incidência de lesão esofágica ([R] 3,96; IC de 95%, 1,94–8,07; p < 0,006).^25^

No estudo OPERA, 200 pacientes foram randomizados para receber ablação com ou sem monitoramento da temperatura esofágica. A incidência geral de LTE foi de 10%, sem diferença estatisticamente significativa entre os grupos. No entanto, entre os pacientes do grupo com monitoramento, nove dos dez que desenvolveram lesão apresentaram temperaturas esofágicas máximas superiores a 39 °C, reforçando a associação entre temperaturas elevadas e dano à mucosa.^26^

Um método alternativo para monitorar alterações térmicas esofágicas é a termografia por infravermelho, que permite avaliação em tempo real por meio de sensores infravermelhos. No estudo HEAT-AF, envolvendo 63 pacientes, aqueles com lesões confirmadas por endoscopia apresentaram temperaturas esofágicas máximas significativamente mais altas (56,3 ± 4,6 °C vs. 45,7 ± 5,6 °C). O aumento da temperatura, medido por termografia por infravermelho, foi preditor de LTE (OR de 1,52 por cada aumento de 1 °C; IC de 95%, 1,14–2,05; p = 0,0008), demonstrando excelente acurácia diagnóstica (AUC = 0,927).^27^

Recentemente, um modelo baseado em inteligência artificial foi desenvolvido para identificar padrões de variação de temperatura associados à lesão esofágica. Em um estudo com 94 pacientes submetidos à ablação por RF ou crioablação, 11 desenvolveram lesões esofágicas (10 após RF e 1 após crioablação). Embora a temperatura máxima absoluta não tenha se correlacionado com lesão nos casos de RF, a taxa de aumento da temperatura no pico térmico foi significativamente maior entre os pacientes que apresentaram lesão (0,08 °C/s vs. 0,02 °C/s; p = 0,033).^28^

A introdução dos cateteres com Força de Contato (FC) reduziu significativamente a taxa de complicações graves associadas à ablação da FA.^29^ Posteriormente, foi desenvolvido o Índice de Ablação (Ablation Index - AI) com o objetivo de otimizar ainda mais a segurança e a durabilidade das lesões, integrando parâmetros como CF, potência e tempo. A eficácia e segurança da ablação guiada por AI versus guiada por CF foram avaliadas em uma coorte de 200 pacientes submetidos à ablação da PPAE, com potência limitada a 20–25 W e sem monitoramento da temperatura esofágica. A FC alvo variou de 5 g a 40 g. Embora não tenha sido observada diferença significativa na incidência geral de LTE (6% no grupo FC vs. 5% no grupo AI), um AI> 520 na PPAE foi identificado como preditor de lesão (OR 3,84; p = 0,039).^30^ As lesões ulcerativas profundas (> 5 mm) foram associadas a AI ≥ 520 (OR 7,05; p = 0,039), à criação de linhas adicionais de ablação no teto ou na PPAE (OR 5,19; p = 0,039), à presença de cardiopatia estrutural e a escores elevados de CHA_2_DS_2_-VASc. No entanto, não foi possível realizar análise multivariada devido ao número limitado de eventos.

Em um estudo realizado por Wolff et al.,^20^ preditores adicionais de lesão esofágica incluíram valores mais altos do AI na PPAE (499 vs. 473; p = 0,04), maior número de pontos de ablação (61,5 vs. 48,2; p = 0,007) e maior força média de contato (15,8 g vs. 13,6 g; p = 0,022).

Um registro brasileiro publicado recentemente demonstrou que o uso de cateteres com ponta de 8 mm não irrigados esteve significativamente associado a um risco aumentado de LTE (OR 3,13; IC de 95% 1,21–8,18; p = 0,019). Entre os 823 pacientes incluídos na análise, o único que evoluiu para uma FAE havia sido submetido à ablação com esse tipo de cateter.^31^

Em resumo, diversos fatores relacionados ao procedimento merecem atenção especial para reduzir o risco de LTE durante a ablação por RF da FA. Deve-se ter cautela diante de temperaturas esofágicas elevadas ou aumentos rápidos de temperatura, bem como na aplicação de FC excessiva ou valores elevados de AI na PPAE. Sempre que possível, recomenda-se o uso de cateteres irrigados com monitoramento de força de contato, enquanto os cateteres não irrigados com ponta de 8 mm devem ser evitados.

### Medidas de proteção para reduzir o risco de lesão térmica esofágica

Nas últimas décadas, a ablação convencional por cateter para FA passou por avanços significativos tanto na técnica quanto na tecnologia, com ênfase em três domínios principais: segurança do paciente, eficácia do procedimento e eficiência operacional.^32^ No que diz respeito à proteção esofágica, diversas estratégias podem ser empregadas para mitigar o risco de LTE. Essas estratégias incluem a seleção cuidadosa da fonte de energia, do tipo de cateter, da abordagem de ablação, além da incorporação de diferentes modalidades de monitoramento voltadas para a avaliação e proteção esofágica em tempo real.

### Fonte de energia para ablação

A primeira decisão crítica antes de realizar a ablação da FA envolve a escolha da fonte de energia apropriada. Entre as modalidades de energia térmica – excluindo a Ablação por Campo Pulsado (Pulsed Field Ablation - PFA), que será discutida separadamente — a ablação por RF parece estar associada a um risco mais elevado de envolvimento esofágico em comparação com a crioablação. No estudo POTTER-AF, a incidência de FAE foi significativamente maior em pacientes tratados com ablação por RF do que com crioablação (0,038% vs. 0,0015%, respectivamente; p < 0,001).^12^

Além disso, em um estudo de coorte envolvendo 236 pacientes, Zhang et al.^33^ demonstraram que a crioablação esteve associada de forma independente a um risco reduzido de lesão esofágica (OR 0,279; IC de 95% 0,108–0,723; p = 0,01).^33^

### Cateter com força de contato e ablação de alta potência e curta duração

Quando a energia por RF é selecionada, o uso de um cateter com FC parece desempenhar um papel fundamental na redução do risco de lesão esofágica. No estudo RESCUE-AF, 89 pacientes foram randomizados para realizar ablação de FA utilizando um cateter com FC (limitado a <20 g) ou um cateter sem FC. Nenhuma LTE foi observada no grupo com cateter FC, enquanto a incidência de LTE no grupo sem CF foi de 20%. Os autores concluíram que limitar a FC durante a ablação pode prevenir efetivamente a lesão esofágica sem comprometer a eficácia do procedimento.^34^

Em relação a estratégias de entrega de energia, a técnica de ablação de alta potência e curta duração (High-Power, Short-Duration - HPSD) tem sido proposta como uma forma de promover o aquecimento eficiente do tecido miocárdico atrial, ao mesmo tempo em que minimiza os danos condutivos às estruturas adjacentes. Em um estudo envolvendo 687 pacientes, a HPSD (50 W por 5 segundos) foi comparada à ablação convencional (≤35 W na PPAE por 10-30 segundos), com a avaliação do envolvimento esofágico realizada por ressonância magnética 24 horas após o procedimento. Nenhum caso de FAE foi relatado, e as taxas de recorrência da FA após 2,5 anos foram semelhantes entre os grupos (42% vs. 41%; p = 0,57).^35^

Outro estudo comparou a HPSD a 45 W por até seis segundos na PPAE com a ablação convencional a 20 W por até 30 segundos na mesma região, utilizando FC de 10-20 g e 10-30 g, respectivamente, em 355 pacientes. O grupo HPSD apresentou tempos significativamente menores de procedimento e aplicação de RF, além de uma taxa mais alta de isolamento de primeira passagem, juntamente com uma elevação reduzida da temperatura esofágica (38,1% vs. 84,5% no grupo convencional; p < 0,0001). Além disso, o grupo HPSD apresentou menor incidência de recorrência da fibrilação atrial (FA) durante o acompanhamento.^36^

No estudo Frankfurt AI-HP ESO-I,^37^ 122 pacientes foram submetidos à ablação utilizando uma combinação de alta potência (50 W) e configurações de IA de 550 para a parede anterior e 400 para a parede posterior, com monitoramento da temperatura esofágica. A avaliação endoscópica foi realizada em pacientes cuja temperatura esofágica excedeu 39 °C. Entre os 57 pacientes com temperaturas esofágicas elevadas, apenas dois (3,5%) apresentaram evidências de LTE e nenhum evoluiu para complicações clinicamente significativas. Os autores sugeriram que aplicações de alta potência e curta duração podem reduzir o risco de lesão esofágica ao limitar o tempo total de aplicação, o aquecimento condutivo e ablações repetidas desnecessárias em áreas não contíguas.^37^

### Monitoramento da temperatura esofágica

Uma das estratégias preventivas mais debatidas na ablação da FA é o uso do monitoramento da temperatura esofágica, o que levou a práticas heterogêneas entre os operadores. O estudo OPERA, mencionado anteriormente, avaliou o monitoramento da temperatura intraesofágica utilizando a sonda multissensor SensiTherm™ e constatou que seu uso não reduziu a incidência de complicações esofágicas.^26^ O estudo sugeriu que as temperaturas máximas registradas pela sonda não se correlacionaram com a ocorrência de lesões esofágicas. No entanto, no grupo sem monitoramento de temperatura, as aplicações de RF na PPAE foram limitadas a 25W, valor significativamente menor em comparação ao grupo com controle de temperatura. Além disso, o único paciente do estudo que necessitou de terapia endoscópica para uma úlcera esofágica profunda pertencia ao grupo sem monitoramento de temperatura.

Outro dispositivo, a sonda multissensor CircaS-Cath™, foi avaliado em 36 pacientes submetidos à ablação por RF para FA. Neste estudo, a entrega de energia era interrompida se a temperatura esofágica aumentasse ≥ 0,2 °C ou ultrapassasse 39 °C. Na endoscopia de acompanhamento, apenas um paciente (3%) apresentou uma lesão esofágica atribuída à lesão térmica, enquanto quatro pacientes (11,1%) apresentaram lesões traumáticas leves. Esses achados sugerem que o CircaS-Cath™ pode estar associado a um baixo risco de LTE quando utilizado para guiar a entrega de energia durante a ablação. No entanto, um editorial subsequente levantou preocupações metodológicas em relação ao estudo, especialmente quanto ao momento da realização da endoscopia e à classificação de certas lesões como mecânicas, e não térmicas, em sua origem.^38,39^

Um terceiro estudo randomizou 106 pacientes para receber monitoramento da temperatura esofágica com um cateter de três sensores (J-Cath™) ou um cateter de cinco sensores (SensiTherm™). Embora o tempo de fluoroscopia tenha sido menor no grupo com cinco sensores, o número total de lesões e o tempo geral de ablação foram maiores. Não foi observada diferença significativa na incidência de LTE entre os grupos (5,8% vs. 7,4%, respectivamente; p = 1,0).^24^

Apesar dos achados inconsistentes quanto à eficácia do monitoramento da temperatura esofágica na prevenção de lesões, dados do registro POTTER-AF sugerem um possível benefício em termos de sobrevida. Entre os pacientes que desenvolveram FAE, apenas 24,6% haviam sido submetidos à ablação com termometria esofágica. No entanto, o uso de uma sonda de temperatura esteve associado de forma independente à melhora na sobrevida (OR 0,231; IC de 95% 0,074–0,724; p = 0,012).^12^ Considerando que os pacientes com controle de temperatura apresentaram temperatura máxima média elevada (40,2 ± 2,2 °C), a vigilância intensificada quanto a sinais ou sintomas nesse grupo pode ter permitido o diagnóstico precoce da FAE, contribuindo potencialmente para melhores desfechos clínicos.

Uma das limitações na avaliação da temperatura esofágica durante a ablação da FA é a considerável variabilidade entre os termômetros, que são convencionalmente posicionados apenas na orientação craniocaudal. Consequentemente, os sensores podem não registrar com precisão as regiões do esôfago com as temperaturas mais elevadas. Para superar essa limitação, Leite et al. propuseram o monitoramento da temperatura esofágica utilizando um cateter de ablação defletível posicionado no local mais próximo da lesão, guiado por ecocardiografia intracardíaca. Em uma análise piloto envolvendo 45 pacientes, com potência limitada a 25W na PPAE e interrupção da ablação caso o aumento de temperatura excedesse >2°C, nenhuma LTE foi observada nas endoscopias realizadas até 48 horas após o procedimento.^40^ O uso de dispositivos defletíveis ou a simples visualização dos sensores por meio da ecocardiografia intracardíaca pode auxiliar na avaliação precisa da proximidade entre a ablação e o esôfago.

### Deslocamento esofágico

Outra estratégia de proteção que tem ganhado atenção é o deslocamento esofágico, que envolve o reposicionamento mecânico do esôfago para longe da zona de ablação, a fim de minimizar a exposição à energia de RF.

O estudo EASY AF^41^ investigou essa abordagem randomizando pacientes submetidos à ablação da FA para receberem o deslocamento esofágico com o dispositivo ESOlution™ (S4 Medical Corp) ou sem deslocamento. O dispositivo combina sucção a vácuo com deflexão mecânica para reposicionar o esôfago em tempo real. O estudo foi encerrado precocemente após a inclusão de 120 pacientes devido à eficácia demonstrada da intervenção. A incidência de LTE foi significativamente menor no grupo com deslocamento em comparação ao grupo controle (5,7% vs. 35,7%; p < 0,0001). A análise multivariada confirmou o deslocamento esofágico como um fator de proteção independente, reduzindo o risco de lesão em 87% (OR 0,13; IC de 95%, 0,04–0,46; p = 0,001).^41^

No entanto, um editorial sobre o estudo EASY AF levantou várias preocupações em relação a essa estratégia. O texto destacou o potencial de distorção da anatomia do AE, especialmente durante procedimentos prolongados, o que pode comprometer a precisão na localização dos óstios das VPs e aumentar o risco de estenose dessas veias. Além disso, o editorial apontou incertezas quanto à avaliação da função do nervo frênico no contexto do deslocamento esofágico, indicando a necessidade de mais investigações.^42^

### Resfriamento esofágico

O resfriamento esofágico também tem sido explorado como uma estratégia preventiva para reduzir o risco de lesão térmica durante a ablação da fibrilação atrial (FA). O resfriamento pode ser realizado por meio de diferentes métodos, incluindo dispositivos dedicados ao resfriamento ou infusão direta via sonda gástrica.

Uma meta-análise de estudos com pequenas amostras, totalizando 294 pacientes, não encontrou diferença significativa na incidência geral de lesão esofágica entre os grupos que receberam resfriamento esofágico e os controles (15% vs. 19%; risco relativo [RR], 0,86; intervalo de confiança [IC] de 95%, 0,31–2,41). No entanto, foi observada uma redução na incidência de lesões esofágicas graves no grupo com resfriamento (1,5% vs. 9%; RR, 0,21; IC 95%, 0,05–0,80). Esse achado, contudo, não foi confirmado após uma análise de sensibilidade que excluiu o estudo que mais contribuía para a heterogeneidade. Foi observada uma variabilidade considerável entre os estudos incluídos quanto aos métodos de resfriamento esofágico, estratégias de monitoramento de temperatura, momento da avaliação endoscópica e os tipos de técnicas e ferramentas de ablação utilizadas. Além disso, a definição de lesão grave variou entre os estudos, o que limita a possibilidade de se tirar conclusões definitivas sobre a eficácia protetora do resfriamento esofágico.^43^

O interesse pelo resfriamento esofágico aumentou recentemente após os resultados de uma ampla análise temporal multicêntrica envolvendo 25 186 pacientes submetidos à ablação por RF para FA em 30 centros. O estudo avaliou o impacto do resfriamento esofágico utilizando o dispositivo ensoETM™ – um sistema de circuito fechado conectado a um trocador de calor que circula água mantida a 4 °C para regular ativamente a temperatura intraluminal do esôfago. Entre 10 962 procedimentos realizados sem resfriamento, foram relatados 16 casos de FAE. Em contraste, nenhum caso de FAE foi observado entre os 14 224 procedimentos em que o resfriamento esofágico com o dispositivo ensoETM™ foi utilizado. Vale destacar que mais de 70% dos procedimentos em ambas as coortes foram realizados utilizando protocolos de HPSD com potência entre 40 e 50 W.^44^

#### Abordagem para prevenção de lesões esofágicas graves durante a ablação por radiofrequência da fibrilação atrial: identificação de pacientes de alto risco

Em centros onde a ablação por RF continua sendo a principal estratégia para o tratamento da FA, a identificação de pacientes de alto risco – assim como o reconhecimento de fatores relacionados ao procedimento que aumentam a probabilidade de LTE – é essencial para a estratificação do risco de progressão para FAE (Figura Central).

**Figura 3:**
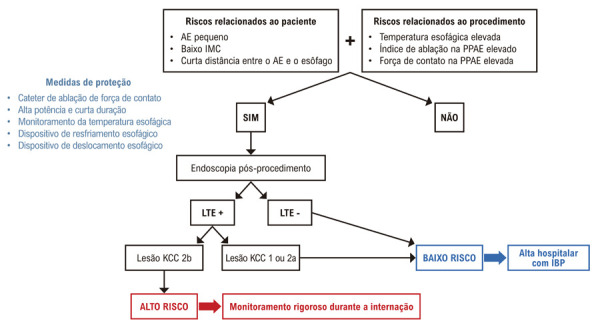
– Fluxograma para identificação e monitoramento de lesão esofágica após ablação por radiofrequência da fibrilação atrial nos casos em que o monitoramento da temperatura esofágica é utilizado; AE: átrio esquerdo; IMC: índice de massa corporal; PPAE: parede posterior do átrio esquerdo; LTE: lesão térmica esofágica; KCC: Classificação de Kansas City; IBP: inibidor da bomba de prótons.

Propomos um fluxograma para a avaliação do envolvimento esofágico após ablação por RF para FA (Figura 3). O uso de cateter com FC deve ser padrão em todos os procedimentos. Além disso, a ablação por HPSD parece oferecer um perfil de segurança favorável. A monitorização da temperatura intraesofágica, amplamente disponível, pode ser considerada uma estratégia de primeira linha devido à sua acessibilidade em comparação com os sistemas de resfriamento esofágico. A visualização dos sensores por meio de ecocardiografia intracardíaca pode auxiliar na melhor avaliação da distância entre o esôfago e o cateter de ablação.

O deslocamento esofágico pode ser empregado seletivamente em cenários de alto risco, como quando a distância entre o átrio esquerdo e o esôfago é mínima ou quando ocorre elevação significativa da temperatura esofágica durante a ablação.

Fatores relacionados ao paciente associados ao aumento do risco de LTE incluem:

- Distância curta entre o AE e o esôfago
- Dimensões menores do AE
- Baixo IMC

Fatores relacionados ao procedimento incluem:

- Temperatura esofágica elevada durante a ablação da PPAE
- Valores elevados de AI e/ou FC

Na presença de uma combinação desses fatores de risco, é razoável realizar uma esofagogastroduodenoscopia entre 24 e 72 horas após a ablação.

- Se o paciente não apresentar fatores de risco, ou se forem identificadas lesões leves [Classificação de Kansas City (KCC) tipos 1 e 2a] na endoscopia de acompanhamento, o paciente é considerado de baixo risco para FAE. Esses indivíduos podem receber alta com prescrição de um inibidor da bomba de prótons.
- Por outro lado, se forem observadas lesões esofágicas profundas (KCC tipo 2b), o paciente deve ser classificado como de alto risco e permanecer hospitalizado para monitoramento rigoroso e intervenção precoce, permitindo o diagnóstico oportuno de uma possível FAE.

#### A ablação por campo pulsado seria a solução para todos os desafios relacionados a lesões esofágicas na ablação da fibrilação atrial?

A PFA representa um avanço significativo no tratamento da FA e já se tornou uma realidade clínica em diversos países. Os resultados iniciais – especialmente em relação à segurança e à redução de lesões em tecidos colaterais – são promissores. A PFA utiliza energia elétrica não térmica, aplicada por meio de eletrodos de cateter, para induzir a eletroporação irreversível, criando microporos nas membranas dos cardiomiócitos que levam à morte celular seletiva, preservando os tecidos não cardíacos adjacentes.

Em três estudos multicêntricos — IMPULSE, PEFCAT e PEFCAT II — envolvendo um total de 121 pacientes com FA paroxística, não foram relatadas complicações esofágicas. A taxa sobrevida livre de arritmia atrial após um ano foi de 84,5% com o uso de ablação otimizada por onda bifásica.^45^

O estudo ADVENT,^46^ um ensaio clínico randomizado e controlado, comparou 305 pacientes tratados com PFA a 302 tratados com ablação térmica convencional. A PFA demonstrou ser não inferior em termos de eficácia e segurança, com taxas de eventos adversos graves de 2,1% no grupo PFA versus 1,5% no grupo térmico.

Uma revisão sistemática e meta-análise de 18 estudos envolvendo 4998 pacientes comparou a PFA com a ablação térmica (radiofrequência e crioablação). A PFA foi associada a uma redução de 83% no risco de lesão esofágica (OR 0,17; IC 95%, 0,06–0,46; p < 0,01). No entanto, a PFA também foi associada a um aumento de quase três vezes no risco de tamponamento cardíaco (OR 2,98; IC 95%, 1,27–7,00; p = 0,01).^47^

Outra meta-análise comparou os desfechos em 497 pacientes tratados com PFA *versus* 1113 pacientes tratados com crioablação em nove estudos. A incidência de eventos adversos maiores foi significativamente menor no grupo PFA (0,4% vs. 5,6%), mesmo após análise de sensibilidade. Esse achado foi principalmente impulsionado por taxas mais altas de paralisia do nervo frênico e complicações vasculares no grupo de crioablação.^48^

O maior registro de segurança da PFA até o momento, o estudo MANIFEST-17K, analisou dados de 17 642 pacientes tratados em 106 centros. Não foram relatados casos de lesão esofágica grave, estenose das veias pulmonares ou paralisia persistente do nervo frênico. Complicações maiores ocorreram em aproximadamente 1% dos casos, incluindo: tamponamento cardíaco (0,36%), complicações vasculares (0,3%), espasmo da artéria coronária (0,14%) e hemólise com insuficiência renal aguda necessitando de diálise (0,03%).^49^

Até o momento, nenhum estudo clínico envolvendo PFA demonstrou lesão esofágica persistente detectável por endoscopia ou técnicas de imagem.^50^ Embora a PFA pareça oferecer proteção substancial ao esôfago e às estruturas adjacentes, complicações emergentes como tamponamento cardíaco, espasmo coronariano, mioglobinúria e lesão renal ainda requerem investigação adicional.

É importante destacar que uma grande proporção das ablações de FA realizadas mundialmente ainda utiliza RF ou crioablação, especialmente em regiões onde a PFA ainda não está amplamente disponível — como na América Latina. Além disso, os dados de longo prazo sobre a segurança e durabilidade da PFA continuam limitados, ressaltando a necessidade de monitoramento contínuo e mais pesquisas.

## Conclusões

A ablação por RF continua sendo um tratamento confiável e amplamente utilizado para FA na prática clínica. No entanto, devido à escassez de dados de longo prazo sobre a PFA e sua disponibilidade limitada em muitas regiões, ainda existem preocupações quanto a complicações esofágicas graves, especialmente a FAE. LTE ocorrem em até aproximadamente um quarto dos casos, sendo influenciadas por fatores do paciente e do procedimento, e exigem classificação adequada, endoscopia baseada em risco e estratégias de proteção.

A implementação de um fluxograma estruturado para avaliação pós-ablação do envolvimento esofágico pode ser importante para a identificação precoce de pacientes de alto risco, permitindo intervenção imediata e aumentando significativamente a segurança do paciente. A adoção desses protocolos de avaliação de risco e monitoramento pode reduzir a ocorrência de complicações potencialmente fatais.
