# Supplementary material for: Complicações Esofágicas Graves após Ablação de Fibrilação Atrial: Ainda Devemos Nos Preocupar?
Source: Arq Bras Cardiol. 2025 Dec 18;122(11):e20250290. [Article in Portuguese] doi: 10.36660/abc.20250290 (PMC12978277; doi:10.36660/abc.20250290)
Supplement: Translation

# Translation: Severe Esophageal Complications Following Atrial Fibrillation Ablation: Should We Still Be Concerned?

**DOI:** 10.36660/abc.20250290i

**Authors:** Anibal Pires Borges, Carlos Kalil, Josep Brugada, José Plutarco Gutiérrez Yanez, Pablo da Costa Soliz, Marco Aurelio Lumertz Saffi, Anderson Donelli da Silveira, Maurício Pimentel

**Affiliations:** 1 Brazil Programa de Pós-Graduação em Ciências Cardiovasculares da Universidade Federal do Rio Grande do Sul, Porto Alegre, RS – Brazil; 2 Brazil Santa Casa de Porto Alegre, Porto Alegre, RS – Brazil; 3 Spain Institut Clínic Cardiovascular (ICCV), Hospital Clínic, Universitat de Barcelona, Institut d’Investigacions Biomèdiques August Pi i Sunyer (IDIBAPS), Barcelona – Spain; 4 Spain Ar-rhythmias Unit, Hospital Sant Joan de Déu, University of Barcelona, Barcelona – Spain

**Keywords:** Atrial Fibrillation, Esophageal Fistula, Postoperative Complications

## Abstract

Atrial fibrillation (AF) ablation has been increasingly employed as an effective strategy for rhythm control, leading to a reduction in arrhythmia burden, improved quality of life, and, in selected cases, decreased mortality. However, as an invasive procedure, it carries inherent risks. Severe esophageal complications-such as esophageal perforation or atrioesophageal fistula- though rare (with an incidence ranging from 0.025% to 0.113%), are associated with significant morbidity and mortality. Multiple risk factors for esophageal injury have been identified, including both patient-related and procedure-related characteristics. To mitigate these risks during radiofrequency (RF) ablation, several preventive measures have been adopted, including the use of contact force catheters, high-power short-duration energy applications, and protective strategies such as esophageal temperature monitoring, displacement devices, and cooling techniques. Deep esophageal lesions detected through surveillance endoscopy are considered potential precursors to more serious complications and, therefore, warrant close attention. Enhanced monitoring of patients with such lesions may be crucial for enabling early diagnosis and timely intervention to prevent the progression to more severe complications. While pulsed field ablation therapy, which poses a lower risk to the esophagus and other surrounding structures, is not yet widely available, the implementation of robust monitoring strategies can significantly improve clinical outcomes and enhance patient safety in the context of RF AF ablation.

## Introduction

Atrial fibrillation (AF) is the most prevalent sustained arrhythmia in adults, associated with significant morbidity and mortality, and imposes a substantial burden on patients, healthcare providers, and health systems worldwide.^1^ Catheter ablation represents a key therapeutic strategy in the management of AF, with superior efficacy in rhythm control compared to antiarrhythmic medications, and evidence of symptomatic improvement, quality of life enhancement,^2-4^ and, in patients with heart failure and reduced ejection fraction, mortality reduction.^5,6^

Despite its benefits, AF ablation involves procedural risks related to vascular access, catheter manipulation, and energy delivery in the left atrium (LA), which is close to thermally sensitive structures. Recent data indicate complication rates from 2.5% to 8%, with a 30-day mortality below 0.1%. Operator experience and higher procedural volumes are associated with fewer complications.^1,7^ Over the years, the complication rates have markedly decreased.^8^ Severe complications of AF ablation include esophageal perforation and atrioesophageal fistula (AEF) (Figure 1), thromboembolic events, cardiac tamponade, pulmonary vein stenosis, phrenic nerve injury, and vascular complications.^1,7^

**Figure 1:** – Esophageal perforation following atrial fibrillation ablation in a patient who developed an atrioesophageal fistula (image by the authors).

Although rare, major esophageal injuries – particularly perforation and AEF – are associated with significant morbidity and mortality.^7^ Understanding the contributing factors to these complications is crucial, especially considering the elective nature of the procedure, which prompted us to conduct a review on the topic. This review included relevant publications indexed in PubMed from 2004 to 2025, using the following keywords: atrial fibrillation ablation, esophageal complications, AEF, and esophageal thermal injuries (ETIs).

### Atrioesophageal fistula: incidence, pathophysiology, and precursor injuries

AEF was first described in 2004, with one case reported by Scanavacca et al.^9^ and two by Pappone et al.^10^ Among these initial cases, two patients died and one required emergent surgical intervention. A Brazilian registry, encompassing 8,863 procedures performed between 2003 and 2015, identified ten cases of AEF (incidence of 0.113%). Of these, seven were fatal and two survivors experienced neurological sequelae.^11^ In the *POTTER-AF study* – the largest registry on this complication to date – 138 cases of AEF were reported among 553,729 ablations, yielding an incidence of 0.025%. The mortality rate was 65.8%, with only 15.4% of patients recovering without sequelae.^12^ Although the incidence reported in the *POTTER-AF* study is lower than that observed in the Brazilian registry, it underscores the devastating clinical impact of this complication despite advances in ablation technology. Additionally, the true incidence of AEF may be underestimated due to limited screening practices in many centers and a lack of comprehensive data from Latin America.^13^

The precise pathophysiology of AEF remains incompletely understood, but ETI due to LA ablation is thought to initiate the cascade leading to esophageal perforation and fistula formation. Proposed mechanisms include direct mucosal injury from thermal energy, exacerbated by gastroesophageal reflux disease and impaired esophageal motility, potentially due to injury of the periesophageal nerve plexus during the procedure or under general anesthesia.^14^

The pulmonary vein (PV) antrum, situated in the posterior wall of the LA (LAPW) and bordered by the four PV ostia, is the primary target during wide antral circumferential ablation. This technique employs radiofrequency energy to create conduction block by inducing muscular discontinuity and electrically isolating the PVs. However, the extensive lesion sets required for effective isolation may inadvertently increase the risk of thermal damage to adjacent structures – particularly the esophagus.^15^

Post-procedural upper endoscopy is a valuable tool for early identification of esophageal involvement, with reported ETI incidence in recent series ranging from 1.2% to 24.3%.^16,17^ ETIs are considered precursor lesions for more severe complications. The most widely adopted classification system for ETIs was proposed by the Kansas City group, based on 30 studies involving 4,473 patients, of whom 3,921 underwent endoscopy or capsule endoscopy within seven days of AF ablation. In this systematic review, the overall incidence of ETIs was 14.5% (570 cases), categorized as follows:^18^ type 1 - erythema; type 2a - superficial ulcer; type 2b - deep ulcer; type 3a - esophageal perforation without AEF; and type 3b - esophageal perforation with AEF (Table 1, Figure 2).

**Table 1:** – Kansas City Classification of Esophageal Injuries Following Atrial Fibrillation Ablation (adapted from Yarlagadda et al., 2019)

Type	Characteristic
**1**	Erythema
**2a**	Superficial ulcer
**2b**	Deep ulcer
**3a**	Perforation without communication with the atria
**3b**	Perforation with atrioesophageal fistula

**Figure 2:** – Examples of Esophageal Thermal Injuries (images by the authors).

Among 428 patients with type 1 or 2a lesions, none progressed to perforation or AEF. In contrast, among 142 patients with type 2b lesions (deep ulcers), six developed perforations: five cases were not associated with AEF (one of which was fatal), and one progressed to AEF (also fatal). A separate registry of 1,802 patients who underwent AF ablation and endoscopic evaluation within seven days post-procedure reported an 18% incidence of ETIs (150 patients). Among these, 98 exhibited erythema or erosions, while 52 presented with ulcers (deep lesions). Progression to esophageal perforation or AEF occurred in five patients—all of whom had deep ulcers previously identified as precursor lesions.^19^ These findings underscore the importance of early identification and close surveillance of patients with deep esophageal lesions, who may benefit from targeted interventions to mitigate the risk of severe complications.

### Risk factors for esophageal thermal injury

Many risk factors for the development of esophageal injury following AF ablation have been reported, although findings have not always been consistent across studies. Some of these factors are patient-related and pre-existing, while others are procedural and only identified during ablation.

### Patient-related risk factors

Several risk factors for ETI have been associated with anatomical features of the patient. In a study involving 238 patients, a shorter LA–esophagus distance was identified as a significant predictor of injury (3.0 mm vs. 4.4 mm; p = 0.012). Additionally, the LA area measured by magnetic resonance imaging (MRI) was smaller in patients who developed esophageal injury (24.92 ± 6.49 cm^2^ vs. 29.53 ± 7.47 cm^2^; p = 0.032), suggesting that reduced LA dimensions may correlate with narrower periesophageal anatomical spaces.^20^

A registry-based study by Ferraz et al.,^17^ involving 677 patients further demonstrated that a larger LA diameter was associated with a lower risk of ETI. Each 1 mm increase in LA diameter corresponded to a 7% reduction in injury incidence (odds ratio [OR], 0.93; 95% confidence interval [CI], 0.88–0.99; p = 0.028).^17^ Similarly, in a cohort of 267 patients who underwent AF ablation without esophageal temperature monitoring and with a power limit of 25 W applied to the left atrial posterior wall (LAPW), a shorter LA–esophagus distance – assessed via computed tomography – was also associated with a higher risk of ETI. The mean LA–esophagus distance in patients who developed injury was 2.0 ± 0.3 mm, compared to 2.5 ± 0.7 mm in those without injury. Multivariate analysis confirmed this distance as the only significant predictor of ETI (p = 0.0176).^21^

The association between body mass index (BMI) and ETI has also been explored. In a study involving 104 patients who underwent AF ablation with a maximum power of 25 W and 30-second applications to the LAPW, the incidence of asymptomatic esophageal or periesophageal nerve injury was 9.6%. A significant association was observed between low BMI and injury risk, with each 1 kg/m^2^ reduction in BMI increasing the risk by 24% (OR, 0.76; 95% CI, 0.59–0.97; P < 0.05). All affected patients had a BMI < 24.9 kg/m^2^.^22^

In contrast, another study found no association between low BMI and esophageal injury in a cohort of 20 patients with BMI < 24.9 kg/m^2^ who underwent ablation using limited energy (maximum 20W on the LAPW), short duration (maximum 20 seconds), and controlled esophageal temperature (≤39°C).^23^ More recently, a randomized study comparing two types of multisensor esophageal temperature probes reported a 6.6% incidence of ETI, with BMI significantly lower in patients who sustained injury compared to those who did not (21.4 ± 2.5 Kg/m^2^ vs. 24.3 ± 3.4 kg/m^2^; p = 0.022).^24^

These findings suggest that certain patient characteristics, identifiable before the procedure, may predispose to an increased risk of esophageal injury. Specifically, patients with lower BMI and those with a smaller or anatomically normal-sized LA may be at higher risk for ETI during AF ablation.

### Procedure-related risk factors

In addition to patient-related factors, several procedure-related variables have been associated with an increased risk of ETI during AF ablation.

Esophageal temperature elevation has been identified as a significant risk factor in patients undergoing radiofrequency (RF) ablation with esophageal temperature monitoring. In a study by Halbfass et al.,^19^ esophageal temperature monitoring was employed in 35% of 832 AF ablation procedures. A maximum esophageal temperature > 40.5 °C was an independent predictor of esophageal injury (OR 4.044; 95% CI 2.049–7.984; p < 0.001). Moreover, when this temperature threshold was reached, the relative risk (RR) for the development of deep ulcers increased to 2.76.^19^

Similarly, in a cohort of 88 patients undergoing AF ablation with temperature-guided power limitation during LAPW lesions, temperatures ≥ 42 °C were associated with a higher incidence of esophageal injury (RR 3.96; 95% CI 1.94–8.07; p < 0.006).^25^

In the *OPERA* study, 200 patients were randomized to receive ablation either with or without esophageal temperature monitoring. The overall ETI incidence was 10%, with no statistically significant difference between groups. However, among patients in the monitoring arm, nine of ten who developed injury had peak esophageal temperatures > 39 °C, reinforcing the association between elevated temperatures and mucosal damage.^26^

An alternative method of monitoring esophageal thermal changes is infrared thermography (IRT), which enables real-time assessment using infrared sensors. In the *HEAT-AF* study involving 63 patients, those with endoscopically confirmed injuries exhibited significantly higher peak esophageal temperatures (56.3 ± 4.6 °C vs. 45.7 ± 5.6 °C). Temperature elevation, as measured by IRT, was predictive of ETI (OR 1.52 per 1 °C increase; 95% CI 1.14–2.05; p = 0.0008), with excellent diagnostic accuracy (area under the curve [AUC] = 0.927).^27^

Recently, an artificial intelligence-based model has been developed to identify temperature variation patterns associated with esophageal injury. In a study of 94 patients undergoing RF or cryoablation, 11 developed esophageal injuries (10 following RF, 1 following cryoablation). Although absolute peak temperature did not correlate with injury in RF cases, the rate of temperature rise at the thermal peak was significantly greater among those with injury (0.08 °C/s vs. 0.02 °C/s; p = 0.033).^28^

The introduction of contact force (CF) catheters has significantly reduced the rate of severe complications related to AF ablation.^29^ Subsequently, the ablation index (AI) was developed to further enhance procedural safety and lesion durability by integrating parameters such as CF, power, and duration. The effectiveness and safety of AI-guided versus CF-guided ablation were evaluated in 200 patients undergoing LAPW ablation with power limited to 20–25 W and without esophageal temperature monitoring. The target CF ranged from 5 g to 40 g. Although no significant difference in overall ETI incidence was observed (6% in the CF group vs. 5% in the AI group), an AI > 520 on the LAPW was found to be a predictor of injury (OR 3.84; p = 0.039).^30^ Specifically, deep ulcerative lesions (> 5 mm) were associated with AI ≥ 520 (OR 7.05; p = 0.039), the creation of additional ablation lines on the roof or LAPW (OR 5.19; p = 0.039), presence of structural heart disease, and elevated CHA_2_DS_2_-VASc scores. However, multivariate analysis could not be performed due to the small number of events.

In a study by Wolff et al.,^20^ additional predictors of esophageal injury included higher AI values on the LAPW (499 vs. 473; p = 0.04), a greater number of ablation points (61.5 vs. 48.2; p = 0.007), and higher mean contact force (15.8 g vs. 13.6 g; p = 0.022).^20^

A recently published Brazilian registry demonstrated that the use of non-irrigated 8 mm-tip catheters was significantly associated with an increased risk of ETI (OR 3.13; 95% CI 1.21–8.18; p = 0.019). Among the 823 patients included in the analysis, the only individual who progressed to an AEF had undergone ablation using this catheter type.^31^

In summary, several procedure-related factors warrant close attention in order to reduce the risk of ETI during radiofrequency RF ablation for AF. Caution should be exercised in the presence of elevated esophageal temperatures or rapid temperature increases, as well as when excessive CF or high AI values are applied to the LAPW. Whenever feasible, the use of irrigated catheters with contact force monitoring is recommended, while non-irrigated 8 mm-tip catheters should be avoided.

### Protective measures to reduce the risk of esophageal thermal injury

Over the past decades, conventional catheter ablation for AF has undergone significant advancements in both technique and technology, with emphasis on three primary domains: patient safety, procedural efficacy, and operational efficiency.^32^ Regarding esophageal protection, several strategies can be employed to mitigate the risk of ETI. These include careful selection of the energy source, catheter type, ablation approach, and the incorporation of various monitoring modalities aimed at real-time esophageal assessment and protection.

### Ablation energy source

The first critical decision prior to performing AF ablation involves selecting the appropriate energy source. Among thermal energy modalities – excluding pulsed field ablation (PFA), which will be discussed separately – RF ablation appears to carry a higher risk of esophageal involvement compared to cryoablation. In the *POTTER-AF* study, the incidence of AEF was significantly greater in patients treated with RF ablation than with cryoablation (0.038% vs. 0.0015%, respectively; p < 0.001).^12^

Additionally, in a cohort study involving 236 patients, Zhang et al.^33^ demonstrated that cryoablation was independently associated with a reduced risk of esophageal injury (OR 0.279; 95% CI, 0.108–0.723; p = 0.01).^33^

### Contact force catheter and high-power short-duration ablation

When RF energy is selected, the use of a CF catheter appears to play a critical role in reducing the risk of esophageal injury. In the *RESCUE-AF* study, 89 patients were randomized to undergo AF ablation using either a CF catheter (restricted to <20 g) or a non-CF catheter. No ETIs were observed in the CF catheter group, whereas the incidence of ETI in the non-CF group was 20%. The authors concluded that limiting CF during ablation may effectively prevent esophageal injury without compromising procedural efficacy.^34^

Regarding energy delivery strategies, the high-power short-duration (HPSD) ablation technique has been proposed to achieve efficient heating of atrial myocardial tissue while minimizing conductive damage to adjacent structures. In a study involving 687 patients, HPSD ablation (50 W for 5 seconds) was compared to conventional ablation (≤35W on the LAPW for 10–30 seconds) Esophageal involvement was assessed by magnetic resonance imaging 24 hours post-procedure. No cases of AEF were reported, and AF recurrence rates at 2.5 years were similar between groups (42% vs. 41%; p = 0.57).^35^

Another study compared HPSD ablation at 45 W for up to six seconds on the LAPW with conventional ablation at 20 W for up to 30 seconds on the LAPW, using a CF of 10–20 g and 10–30 g, respectively, in 355 patients. The HPSD group demonstrated significantly shorter procedure and RF application times, as well as a higher rate of first-pass isolation, along with a reduced elevation in esophageal temperature (38.1% vs. 84.5% in the conventional group; p < 0.0001). Additionally, the HPSD group showed a lower incidence of AF recurrence during follow-up.^36^

In the *Frankfurt AI-HP ESO-I* study,^37^ 122 patients underwent ablation using a combination of high power (50 W) and AI settings of 550 for the anterior wall and 400 for the posterior wall, with esophageal temperature monitoring. Endoscopic evaluation was performed in patients whose esophageal temperature exceeded 39 °C. Among the 57 patients with elevated esophageal temperatures, only two (3.5%) exhibited evidence of ETI, and none progressed to clinically significant complications. The authors suggested that high-power, short-duration applications may reduce the risk of esophageal injury by limiting overall application time, conductive heating, and unnecessary repeat ablations in non-contiguous areas.^37^

### Esophageal temperature monitoring

One of the most debated preventive strategies in AF ablation is the use of esophageal temperature monitoring, which has led to heterogeneous practices among operators. The previously mentioned *OPERA* study evaluated intraesophageal temperature monitoring using the multi-sensor SensiTherm™ probe and found that its use did not reduce the incidence of esophageal complications.^26^ The study suggested that peak temperatures recorded by the probe did not correlate with the occurrence of esophageal lesions. However, in the group without temperature monitoring, RF applications in the LAPW were limited to 25W, which was significantly lower compared to the group with temperature control. Additionally, the only patient in the study who required endoscopic therapy for a deep esophageal ulcer belonged to the group without temperature monitoring.

Another device, the CircaS-Cath™ multi-sensor probe, was assessed in 36 patients undergoing RF ablation for AF. In this study, energy delivery was interrupted if esophageal temperature increased by ≥ 0.2 °C or exceeded 39 °C. On follow-up endoscopy, only one patient (3%) exhibited an esophageal lesion attributed to thermal injury, while four patients (11.1%) had minor traumatic lesions. These findings suggest that the CircaS-Cath™ may be associated with a low risk of ETI when used to guide energy delivery during ablation. However, a subsequent editorial raised methodological concerns regarding the study, particularly the timing of endoscopy and the classification of certain lesions as mechanical rather than thermal in origin.^38,39^

A third study randomized 106 patients to receive esophageal temperature monitoring with either a three-sensor catheter (J-Cath™) or a five-sensor catheter (SensiTherm™). While fluoroscopy time was shorter in the five-sensor group, the total number of lesions and overall ablation time were greater. No significant difference in ETI incidence was observed between the groups (5.8% vs. 7.4%, respectively; p = 1.0).^24^

Despite inconsistent findings regarding the efficacy of esophageal temperature monitoring in preventing injury, data from the *POTTER-AF* registry suggest a potential survival benefit. Among patients who developed AEF, only 24.6% had undergone ablation with esophageal thermometry. However, the use of a temperature probe was independently associated with improved survival (OR 0.231; 95% CI 0.074–0.724; p = 0.012).^12^ Given that patients with temperature control had an elevated mean maximum temperature (40.2 ± 2.2°C), increased surveillance for signs or symptoms in this group may have enabled earlier diagnosis of AEF, thereby potentially improving patient outcomes.

One of the limitations in assessing esophageal temperature during AF ablation is the considerable variability among thermometers, which are conventionally positioned only in a craniocaudal orientation. Consequently, sensors may not accurately document the esophageal regions with the highest temperatures. To address this limitation, Leite et al. proposed monitoring esophageal temperature using a deflectable ablation catheter placed at the site closest to the lesion, guided by intracardiac echocardiography. In a pilot analysis involving 45 patients, with power limited to 25W in the LAPW and ablation interrupted if temperature increases exceeded >2°C, no ETI were observed on endoscopies performed within 48 hours post-procedure.^40^ The use of deflectable devices or solely visualizing the sensors via intracardiac echocardiography may assist in accurately assessing the proximity of the ablation to the esophagus.

### Esophageal displacement

Another protective strategy that has gained attention is esophageal displacement, which involves mechanically repositioning the esophagus away from the ablation zone to minimize exposure to RF energy.

The *EASY AF* study^41^ investigated this approach by randomizing patients undergoing AF ablation to receive esophageal displacement using the ESOlution™ device (S4 Medical Corp) or no displacement. The device combines vacuum suction with mechanical deflection to achieve real-time repositioning of the esophagus. The study was terminated early after the enrollment of 120 patients due to the demonstrated efficacy of the intervention. The incidence of ETI was significantly lower in the displacement group compared to the control group (5.7% vs. 35.7%; p < 0.0001). Multivariate analysis confirmed esophageal displacement as an independent protective factor, reducing the risk of injury by 87% (odds ratio [OR] 0.13; 95% CI, 0.04–0.46; p = 0.001).^41^

However, an editorial commentary on the *EASY AF* trial raised several concerns regarding this strategy. It highlighted the potential for distortion of LA anatomy, particularly during prolonged procedures, which may compromise the accuracy of PV ostia localization and increase the risk of PV stenosis. Additionally, the editorial noted uncertainty surrounding phrenic nerve function assessment in the context of esophageal displacement, warranting further investigation.^42^

### Esophageal cooling

Esophageal cooling has also been explored as a preventive strategy to reduce the risk of thermal injury during AF ablation. Cooling can be achieved through various methods, including dedicated cooling devices or direct infusion via a gastric tube.

A meta-analysis of small-sample studies totaling 294 patients found no significant difference in the overall incidence of esophageal injury between groups receiving esophageal cooling and controls (15% vs. 19%; RR, 0.86; 95% CI, 0.31–2.41). However, a reduction in the incidence of severe esophageal injuries was observed in the cooling group (1.5% vs. 9%; RR, 0.21; 95% CI, 0.05–0.80). This result, however, was not confirmed after a sensitivity analysis excluding the study contributing most to heterogeneity. Variability was observed among the included studies with respect to esophageal cooling methods, temperature monitoring strategies, timing of endoscopic assessment, and the types of ablation techniques and tools employed. Additionally, the definition of severe injury varied across studies, limiting the ability to draw definitive conclusions regarding the protective efficacy of esophageal cooling.^43^

Interest in esophageal cooling has recently increased following the results of a large multicenter temporal analysis involving 25,186 patients who underwent RF ablation for AF across 30 centers. The study assessed the impact of esophageal cooling using the ensoETM™ device, a closed-loop system connected to a heat exchanger that circulates water maintained at 4 °C to actively control intraluminal esophageal temperature. Among 10,962 procedures performed without cooling, 16 cases of AEF were reported. In contrast, no cases of AEF occurred among the 14,224 procedures in which esophageal cooling with the ensoETM™ device was employed. Notably, over 70% of procedures in both cohorts were performed using AAPCD protocols (40–50 W).^44^

#### Approach to preventing severe esophageal injury during radiofrequency atrial fibrillation ablation: identification of high-risk patients

In centers where RF ablation remains the predominant strategy for AF, identifying high-risk patients – as well as recognizing procedural factors that increase the likelihood of ETI – is essential for stratifying the risk of progression to AEF (Central Figure).

We propose a flowchart for the assessment of esophageal involvement following RF AF ablation (Figure 3). The use of a CF catheter should be standard in all procedures. Additionally, HPSD ablation appears to offer a favorable safety profile. Intraesophageal temperature monitoring, which is widely available, may be considered a first-line strategy due to its accessibility compared to esophageal cooling systems. Visualization of the sensors via intracardiac echocardiography may assist in better assessing the distance between the esophagus and the ablation catheter.

**Figure 3:** – Flowchart for the identification and monitoring of esophageal injury following radiofrequency atrial fibrillation ablation in cases where esophageal temperature monitoring is employed; LA: left atrium; BMI: body mass index; LAPW: left atrial posterior wall; ETI: esophageal thermal injury; KCC: Kansas City Classification; PPI: proton pump inhibitor.

Esophageal displacement may be selectively employed in high-risk scenarios, such as when the LA-esophagus distance is minimal or when significant esophageal temperature elevation occurs during ablation.

Patient-related factors associated with increased risk of ETI include:

- Short LA–esophagus distance
- Smaller LA dimensions
- Low body mass index

Procedure-related risk factors include:

- Elevated esophageal temperature during ablation of the LAPW
- High AI and/or CF values

In the presence of a combination of these risk factors, it is reasonable to perform esophagogastroduodenoscopy between 24 and 72 hours after ablation.

- If the patient presents no risk factors, or if mild lesions (Kansas City Classification [KCC] types 1 and 2a) are identified on follow-up endoscopy, the patient is considered at low risk for AEF. These individuals may be discharged with a prescription for a proton pump inhibitor.
- Conversely, if deep esophageal lesions (KCC 2b) are present, the patient should be classified as high risk and remain hospitalized for close monitoring and early intervention to allow for prompt diagnosis of potential AEF.

#### Could Pulsed Field Ablation be the solution to all esophageal injury-related challenges in AF ablation?

PFA represents a significant advancement in the field of AF ablation and has already become a clinical reality in several countries. Initial results – particularly in terms of safety and the reduction of collateral tissue injury – are promising. PFA employs non-thermal electrical energy delivered via catheter electrodes to induce irreversible electroporation, creating micropores in cardiomyocyte membranes that lead to selective cell death while sparing adjacent non-cardiac tissues.

In three multicenter studies – *IMPULSE*, *PEFCAT*, and *PEFCAT II* – involving a total of 121 patients with paroxysmal AF, no esophageal complications were reported. Freedom from atrial arrhythmia at one year was 84.5% following optimized biphasic waveform ablation.^45^

The *ADVENT* trial,^46^ a pivotal randomized controlled study, compared 305 patients treated with PFA to 302 treated with conventional thermal ablation. PFA was shown to be non-inferior in both efficacy and safety, with serious adverse event rates of 2.1% in the PFA group versus 1.5% in the thermal group.

A systematic review and meta-analysis of 18 studies including 4,998 patients compared PFA to thermal ablation (RF and cryoablation). PFA was associated with an 83% reduction in esophageal injury risk (OR 0.17; 95% CI, 0.06–0.46; p < 0.01). However, PFA was also associated with a nearly threefold increase in the risk of cardiac tamponade (OR 2.98; 95% CI, 1.27–7.00; p = 0.01).^47^

Another meta-analysis compared outcomes in 497 patients treated with PFA versus 1,113 patients treated with cryoablation across nine studies. The incidence of major adverse events was significantly lower in the PFA group (0.4% vs. 5.6%), even after sensitivity analysis. This finding was primarily driven by higher rates of phrenic nerve palsy and vascular complications in the cryoablation group.^48^

The largest PFA safety registry to date, the *MANIFEST-17K* study, analyzed data from 17,642 patients treated across 106 centers. No cases of severe esophageal injury, pulmonary vein stenosis, or persistent phrenic nerve paralysis were reported. Major complications occurred in approximately 1% of cases, including: cardiac tamponade (0.36%), vascular complications (0.3%), coronary artery spasm (0.14%), hemolysis with acute renal failure requiring dialysis (0.03%).^49^

To date, no clinical studies involving PFA have demonstrated persistent esophageal injury detectable via endoscopy or imaging techniques.^50^ While PFA appears to offer substantial protection to the esophagus and surrounding structures, emerging complications such as cardiac tamponade, coronary spasm, myoglobinuria, and renal injury require further investigation.

Importantly, a large proportion of AF ablations worldwide are still performed using RF or cryoablation, particularly in regions where PFA is not yet widely available—such as Latin America. Moreover, long-term data on the safety and durability of PFA remain limited, highlighting the need for continued surveillance and research.

## Conclusions

RF ablation remains a reliable and widely used treatment for AF in clinical practice. However, due to limited long-term data on PFA and its limited availability in many regions, concerns about severe esophageal complications, particularly AEF, still persist. Esophageal thermal injuries occur in up to approximately one quarter of cases, influenced by patient and procedural factors, and require proper classification, risk-based endoscopy, and protective strategies.

Implementing a structured flowchart for post-ablation assessment of esophageal involvement may be important for early identification of high-risk patients, enabling prompt intervention and significantly enhancing patient safety. The adoption of these risk assessment and monitoring protocols can reduce the occurrence of life-threatening complications.
